# Measurement of Pipe and Liquid Parameters Using the Beam Steering Capabilities of Array-Based Clamp-On Ultrasonic Flow Meters

**DOI:** 10.3390/s22145068

**Published:** 2022-07-06

**Authors:** Jack Massaad, Paul L. M. J. van Neer, Douwe M. van Willigen, Michiel A. P. Pertijs, Nicolaas de Jong, Martin D. Verweij

**Affiliations:** 1 Laboratory of Medical Imaging, Department of Imaging Physics, Delft University of Technology, Lorentzweg 1, 2628 CJ Delft, The Netherlands; paul.vanneer@tno.nl (P.L.M.J.v.N.); nicolaas.dejong@tudelft.nl (N.d.J.); m.d.verweij@tudelft.nl (M.D.V.); 2 Netherlands Organisation for Applied Scientific Research (TNO), Oude Waalsdorperweg 63, 2597 AK The Hague, The Netherlands; 3 Electronic Instrumentation Laboratory, Department of Microelectronics, Delft University of Technology, Mekelweg 4, 2628 CD Delft, The Netherlands; d.m.vanwilligen@tudelft.nl (D.M.v.W.); m.a.p.pertijs@tudelft.nl (M.A.P.P.); 4 Thorax Center, Department of Biomedical Engineering, Erasmus Medical Center, Doctor Molewaterplein 40, 3015 GD Rotterdam, The Netherlands

**Keywords:** beam alignment, clamp-on ultrasonic flow meter, self-calibration, transducer arrays

## Abstract

Clamp-on ultrasonic flow meters (UFMs) are installed on the outside of the pipe wall. Typically, they consist of two single-element transducers mounted on angled wedges, which are acoustically coupled to the pipe wall. Before flow metering, the transducers are placed at the correct axial position by manually moving one transducer along the pipe wall until the maximum amplitude of the relevant acoustic pulse is obtained. This process is time-consuming and operator-dependent. Next to this, at least five parameters of the pipe and the liquid need to be provided manually to compute the flow speed. In this work, a method is proposed to obtain the five parameters of the pipe and the liquid required to compute the flow speed. The method consists of obtaining the optimal angles for different wave travel paths by varying the steering angle of the emitted acoustic beam systematically. Based on these optimal angles, a system of equations is built and solved to extract the desired parameters. The proposed method was tested experimentally with a custom-made clamp-on UFM consisting of two linear arrays placed on a water-filled stainless steel pipe. The obtained parameters of the pipe and the liquid correspond very well with the expected (nominal) values. Furthermore, the performed experiment also demonstrates that a clamp-on UFM based on transducer arrays can achieve self-alignment without the need to manually move the transducers.

## 1. Introduction

**Figure 1:**
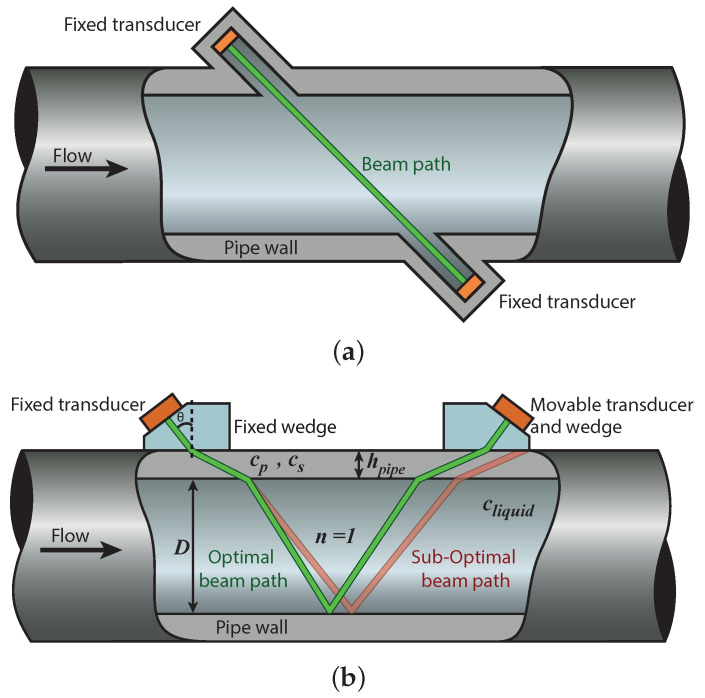
(**a**) In-line and (**b**) clamp-on UFM configuration, which shows that the center of the optimal beam path coincides with the center of the transducer.

Ultrasonic flow meters (UFMs) are used in many applications [1,2]. They consist of one or more pairs of single-element transducers, and are classified into two categories: in-line and clamp-on. In the former, a pipe section is perforated to place the transducers in direct contact with the fluid (Figure 1a). In the latter, the transducers are located on the outer surface of the pipe wall (Figure 1b) [3]. In contrast to in-line UFMs, clamp-on UFMs can be installed without stopping the flow, and operate without perturbing the flow. In addition, they do not puncture the pipe wall, thus maximizing safety. Moreover, clamp-on UFMs may be used on pipes with different dimensions.

The single-element transducers of a conventional clamp-on UFM are placed on a wedge, with angle θ relative to the normal of the wedge–pipe wall interface (see Figure 1b), acoustically coupled to the outer surface of the pipe wall. The transducer generates a compressional wave that is refracted at the interface between the wedge and the pipe wall. The angle of the wedge is sufficiently high such that the incident compressional wave is mode-converted/refracted into a propagating shear wave and an evanescent compressional wave. In the context of clamp-on UFMs, shear waves in pipe walls have typical sound speeds that are closer to the sound speed of the liquid compared to compressional waves; thus, shear waves in the pipe wall refract under higher angles into the liquid compared to the compressional waves in the pipe wall. Waves are transmitted in upstream and downstream directions. The propagation velocity of the waves is affected vectorially by the flow speed ($v_{f}$), leading to a transit time difference (Δt) between the upstream and downstream propagating waves. This Δt is proportional to the flow speed $v_{f}$ [4,5,6].

However, before flow metering, the sensor needs to be aligned: the movable transducer must be manually placed at the correct position on the pipe wall to optimally receive the beam generated by the fixed transducer. It can be seen in Figure 1b that the optimal beam path depends on several parameters of the pipe and the liquid, such as the compressional and shear bulk wave sound speeds of the pipe wall ($c_{p}$ and $c_{s}$, respectively), the pipe wall thickness ($h_{pipe}$), the pipe inner diameter (*D*), the sound speed of the liquid ($c_{liquid}$), and the number of bounces (v-shaped reflections) of the acoustic beam within the pipe wall (*n*). Manual calibration is a cumbersome and time-consuming process for the operator, especially in difficult-to-access places. Moreover, changes in pressure, temperature, or the properties of the flowing liquid/gas itself may change these parameters, causing the acoustic beam to shift from its optimal (calibrated) travel path. This lowers the signal-to-noise ratio (SNR) and introduces errors in the measured flow speed. Furthermore, most of the parameters mentioned above, which are also required for computing the flow speed, are not precisely known in practice.

Transducer arrays generate steered acoustic beams by implementing time delays on the input signals of the individual transducer array elements [7,8,9]. A clamp-on UFM based on two transducer arrays, as shown in Figure 1b, could exploit this capability to achieve correct alignment of the acoustic beams without the need to manually move the transducers. Furthermore, to increase the accuracy of the computed flow speed, the properties of the pipe and the liquid could also be measured using the same transducers.

Different methods have been reported to measure the properties of a pipe using ultrasound. Electromagnetic acoustic transducers (EMATs) have been used to measure pipe diameter and wall thickness. In [10], a method is proposed to measure the pipe diameter, which consists of an EMAT rotating around the outer surface of the pipe wall while emitting acoustic waves and measuring the reflected echoes. In the context of clamp-on UFMs, this is not a practical method to implement. In [11], a pulse-echo technique is proposed to measure the pipe wall thickness. However, both techniques of [10,11] are implemented on a fully submerged pipe, which is not the situation of clamp-on UFMs. Moreover, it is desired to measure the pipe parameters with the same transducers used to measure the flow speed, rather than with the addition of more transducers. Another pulse-echo method to determine the thickness of solids is described in [12]. The method is implemented in a non-submerged configuration, and it takes into account all the potential measurement uncertainties. Such a technique may be feasible in the context of clamp-on UFMs. However, any technique based on pulse-echo will present limitations on pipes of small wall thickness compared to the wavelength of the acoustic beam.

Guided waves have also been used to obtain information regarding the medium within which they propagate. In [13], a method is reported that excites guided waves on a pipe wall and compares the measured dispersion curves with theoretical ones to obtain the pipe diameter and wall thickness. The work of [14,15] shows the use of guided waves to measure the thickness of composite materials. Guided wave tomography has been used to obtain thickness maps of plates and pipe walls [16,17].

In [18,19], it is proposed to measure the sound speed of the liquid inside a pipe using pulse-echo in a similar configuration to that shown in Figure 1b, but a priori information about the pipe wall is needed to compensate for its effect on the measured signals. In [20], it was shown that transducer array-based clamp-on UFMs may use guided waves to invert for the necessary parameters of the pipe and the liquid without any a priori information. However, procedures involving guided waves to obtain the parameters of a waveguide are complex due to the dispersive nature of this kind of wave and the relatively large amount of potential wave modes that may be excited. In the context of clamp-on UFMs, these factors may lead to errors in the obtained parameters of the pipe and the liquid, and thus in the computed flow speeds.

In this work, a simpler method is proposed to obtain the following parameters of the pipe and the liquid: the pipe wall thickness ($h_{pipe}$), the pipe inner diameter (*D*), the relevant pipe wall bulk wave sound speed ($c_{pipe}$), and the sound speed of the liquid ($c_{liquid}$). In addition, it is also shown that a clamp-on UFM based on transducer arrays can be self-calibrated without the need to manually move the transducers. The rest of the paper is structured as follows. Section 2 describes the method to invert for the relevant pipe and liquid parameters. Section 3 describes the experiment performed to test the proposed method. Section 4 shows the experimental results. Section 5 describes the most relevant discussion points. Section 6 summarizes the conclusions.

## 2. Pipe and Liquid Parameters

In this section, a method is described to obtain the relevant parameters required to measure flow using transducer array-based clamp-on UFMs quantitatively and without manual calibration.

**Figure 2:**
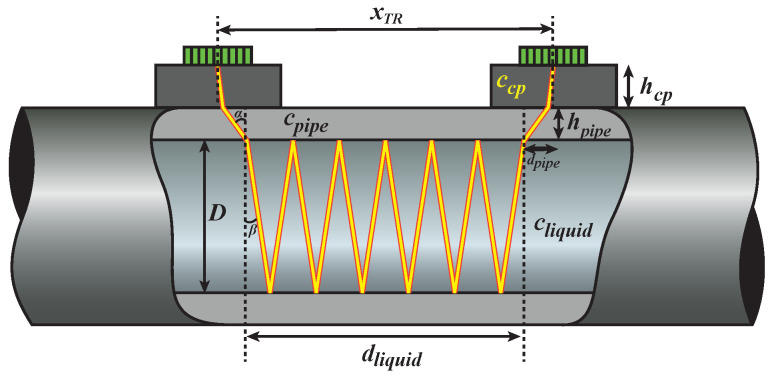
Travel path of an acoustic beam during clamp-on ultrasonic flow measurements. The axial travel path length of the beam is equal to the center-to-center array distance $x_{TR}$. For this particular travel path, n=6.

During flow metering in a clamp-on configuration, an acoustic beam may propagate through a travel path such as the one depicted in Figure 2, and the flow speed $v_{f}$ may be computed by solving the following second-order equation (see Appendix A):

(1) $$\left[\Delta t\sin^{2}\left(\beta \right)\right]v_{f}^{2}+\left[4nD\tan\left(\beta \right)\right]v_{f}-\Delta tc_{liquid}^{2}=0,$$

As shown in Equation (1), in addition to the measured Δt, some parameters of the pipe and the liquid are also needed to compute the flow speed $v_{f}$. These parameters are: the number of bounces (v-shaped reflections) of the beam inside the pipe (*n*), the inner diameter of the pipe (*D*), the sound speed of the liquid ($c_{liquid}$), and the refraction angle of the beam into the liquid (β, see Figure 2), which is a function of the compressional bulk wave sound speed of the coupling piece ($c_{cp}$) and of the bulk wave sound speed of the pipe wall ($c_{pipe}$). The value of $v_{f}$ obtained via Equation (1) is an average of the flow speed profile.

For each number of v-shaped reflections *n* of the acoustic beam within the pipe, the acoustic beam must cover the same axial distance $x_{TR}$, which is known in practice. From Figure 2, it can be seen that this distance is the sum of the axial propagation distance of the acoustic beam through the coupling piece ($d_{cp}$), the pipe wall ($d_{pipe}$), and the liquid ($d_{liquid}$):

(2) $$x_{TR}=d_{cp}+d_{pipe}+d_{liquid}$$

From the geometry of Figure 2, the terms in the right-hand side of Equation (2) may be written as a function of the beam steering angle in the coupling piece (θ, see Figure 1b), the thickness of the coupling piece and the pipe wall ($h_{cp}$ and $h_{pipe}$, respectively), the compressional bulk wave sound speed of the pipe wall, of the coupling piece, and of the liquid ($c_{pipe}$, $c_{cp}$ and $c_{liquid}$, respectively), the number of bounces (v-shaped reflections) of the beam inside the pipe wall (*n*), and the inner diameter of the pipe (*D*):

(3) $$\begin{array}{c} d_{cp}=2h_{cp}\tan\theta ,\\ d_{pipe}=2h_{pipe}\tan\alpha ,\\ d_{liquid}=2nD\tan\beta , \end{array}$$

with:

(4) $$\begin{array}{c} \alpha =\arcsin\left(\frac{c_{pipe}}{c_{cp}}\sin\theta \right),\\ \beta =\arcsin\left(\frac{c_{liquid}}{c_{cp}}\sin\theta \right). \end{array}$$

In Equation (4), α and β represent the propagation angle of the acoustic beam in the pipe wall and in the liquid, respectively (see Figure 2). For five different travel paths, and using Equations (2) and (3), a system of five equations with five unknowns ($h_{pipe}$, $c_{pipe}$, *i*, *D*, and $c_{liquid}$) may be built:

(5) $$\left\{\begin{array}{c} x_{TR}=2h_{cp}\tan\theta_{i}+2h_{pipe}\tan\alpha_{i}+2iD\tan\beta_{i}\\ x_{TR}=2h_{cp}\tan\theta_{i+1}+2h_{pipe}\tan\alpha_{i+1}+2\left(i+1\right)D\tan\beta_{i+1}\\ x_{TR}=2h_{cp}\tan\theta_{i+2}+2h_{pipe}\tan\alpha_{i+2}+2\left(i+2\right)D\tan\beta_{i+2}\\ x_{TR}=2h_{cp}\tan\theta_{i+3}+2h_{pipe}\tan\alpha_{i+3}+2\left(i+3\right)D\tan\beta_{i+3}\\ x_{TR}=2h_{cp}\tan\theta_{i+4}+2h_{pipe}\tan\alpha_{i+4}+2\left(i+4\right)D\tan\beta_{i+4} \end{array}\right.$$

In Equation (5), *i* indicates the minimum number of v-shapes considered in our system of equations, and the indices of the angles θ, α, and β refer to the number of v-shapes *n* for which a specific equation applies. Equations (3) and (5) show a nonlinear relation between the five unknowns. To obtain these parameters, the following procedure is executed: first, the angle θ associated with each travel path is introduced in Equations (3) and (5); second, an initial solution to Equation (5) is provided; third, a nonlinear least squares solver is implemented in MATLAB (The MathWorks, Inc., Nattick, MA, USA) to obtain the final solution.

## 3. Experiment

### 3.1. Setup

**Figure 3:**
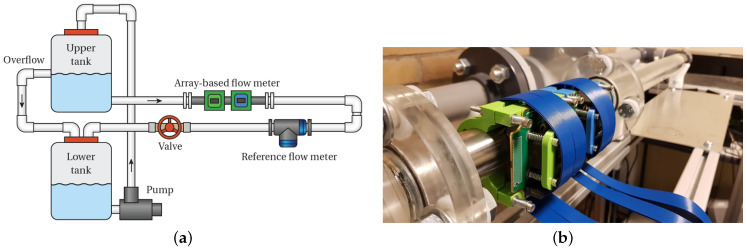
(**a**) Schematic of the custom-made flow loop to test the array-based sensor and perform beam auto-alignment measurements. (**b**) Pipeline section showing the array-based flow meter consisting of two 36-element linear transducer arrays. A lead piece (not visible here) was used as coupling material between the transducer arrays and the stainless steel pipe wall.

A custom-made flow loop was built and filled with water (see Figure 3a). It consists of a pipe with a 40mm inner diameter, including a reference in-line UFM (Optosonics 3400, KROHNE Nederland B.V., Dordrecht, The Netherlands). On the center of a 300mm-long, 1mm-thick, 304 stainless steel pipe section (nominal compressional and shear bulk sound speeds: $c_{p}=5920\;m/s$ and $c_{s}=3141\;m/s$, respectively), a custom-made clamp-on UFM [21] was mounted (see Figure 3b). The sensor consisted of two 36-element linear arrays, with a center-to-center distance of $x_{TR}=80\;\mathrm{mm}$. The arrays consisted of piezo-elements of PZ26 (Meggit A/S, Kvistgård, Denmark) with dimensions of 0.62mm (in azimuth) by 12mm (in elevation), an element pitch of 0.72mm, and a resonance frequency of 1MHz. Per array, a lead piece ($c_{cp}=c_{lead}=2163\;m/s$) was used as an acoustic coupler between the transducers and the pipe wall. The coupler had a flat surface area at the top (coupled to the transducer array), a curved surface area at the bottom (coupled to the pipe wall), and a minimum thickness of $h_{cp}=h_{lead}=11\;\mathrm{mm}$. A Verasonics Vantage 256 system (Verasonics Inc., Kirkland, WA, USA) was used to excite and read out each individual element of the transducer arrays.

### 3.2. Measurement Procedure

Measurements were performed at zero flow conditions. Both transducers were alternately used as transmitters and receivers. A one-cycle sine wave was used as an input signal. Time delays of the input signal were implemented on each transducer array element to generate steered acoustic beams in transmission. A sweep of the beam steering angle in the coupling piece was performed within the range $7^{\circ }\leq \theta \leq 20^{\circ }$, with an angular step of $\Delta \theta =\;0.05^{\circ }$. Within this range, it was expected to encounter the optimal angle of the five travel paths associated with *n* = 4–8 v-shapes of the acoustic beam within the pipe, at which maximum amplitude upon reception was expected since the center of the impinging acoustic beam coincided with the center of the receiving transducer array. Per angle, 1000 repeated acquisitions were recorded, and for different transit time windows (i.e., different travel paths of the acoustic beam), two parameters were monitored: the amplitude of the received upstream and downstream acoustic beams, and the transit time difference Δt between them.

## 4. Results

**Figure 4:**
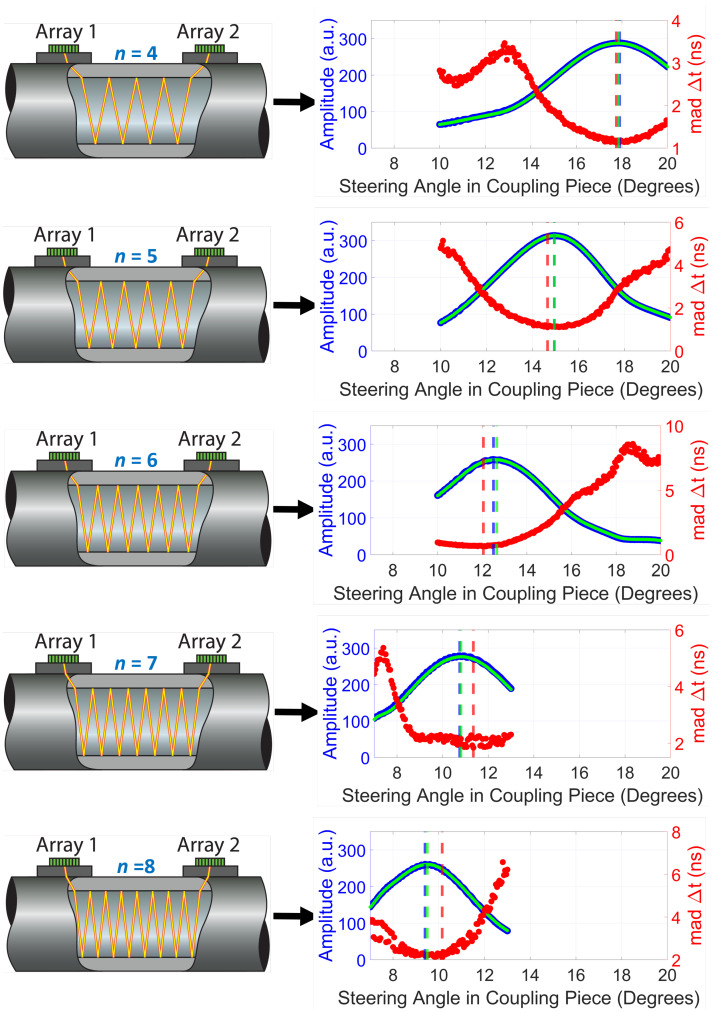
Beam alignment measurements in the context of a transducer array-based clamp-on UFM. On the **left** is shown a sketch of the travel path on which the acoustic beam is being aligned. On the **right** is shown, as a function of the steering angle of the acoustic beam in the coupling piece, the monitored amplitude of the upstream and downstream beams upon reception (in blue and green, respectively) and the computed uncertainty (i.e., mean absolute deviation) of the transit time difference between them (in red). Since the measured upstream and downstream amplitudes are very similar, a slightly different line width is used in the plots to show both amplitudes. The blue and green dashed vertical lines mark the angle that reported maximum amplitude for the upstream and downstream beam, respectively. The red dashed line marks the angle that reported minimum uncertainty of the transit time difference.

Figure 4 shows the acoustic beam amplitudes and the uncertainty (i.e., mean absolute deviation, *mad*) of the computed Δt for five different monitored travel paths (associated with *n* = 4–8) as a function of the steering angle of the acoustic beam in the coupling piece. The *mad* was computed as:

(6) $$mad=\frac{1}{N}\sum_{i=1}^{N}∥x_{i}-\mu ∥,$$

where $x_{i}$ represents an individual transit time difference measurement, and μ represents the mean value of all N=1000 measurements.

It can be observed in Figure 4 that a clear amplitude maximum was found for each travel path. Moreover, there was a negligible visual discrepancy between the upstream and downstream acoustic beams. Such clear amplitude maxima were expected because this parameter is a direct indicator of the signal level. In contrast, the uncertainty of Δt shown in Figure 4 reflects the noise level of the entire system, which makes it a sub-optimal parameter to identify the optimal beam steering angle.

Table 1 summarizes the measured optimal angles for each considered travel path, $\theta_{meas}$, and contrasts them with the angles expected theoretically, $\theta_{theo}$. The very good agreement between both angles demonstrates the suitability of transducer arrays for achieving automatic beam alignment of the transmitting acoustic beams in the context of clamp-on ultrasonic flow metering without having to manually move the transducers along the pipe wall.

It was of interest to investigate the sensitivity of the final solution of Equation (5) to the accuracy of the optimal angles and to the initial solution. To achieve this, the theoretical optimal angles shown in Table 1 (i.e., $\theta_{theo}$) were input into Equation (5). Moreover, a range of initial solutions was defined to be within the limits of practically expected values: $0.5\;\mathrm{mm}\leq h_{pipe}\leq 1.5\;\mathrm{mm}$ in steps of $\Delta h_{pipe}=0.1\;\mathrm{mm}$; $5500\;m/s\leq c_{pipe}\leq 6500\;m/s$ in steps of $\Delta c_{pipe}=20\;m/s$; 2≤i≤5 in steps of Δi=1; 35mm≤D≤45mm in steps of ΔD=0.5mm; and $1300\;m/s\leq c_{liquid}\leq 1700\;m/s$ in steps of $\Delta c_{liquid}=10\;m/s$. For each initial solution, the solver was executed, and a final solution and its associated error were obtained. The optimal solution was considered to be the one that reported the minimum error.

Considering $\theta_{theo}$ and the initial solution space defined above, it was found that all five parameters of the pipe and the liquid ($h_{pipe}$, $c_{pipe}$, *i*, *D*, $c_{liquid}$) reported by the optimal solution were equal to their nominal value, reported in Table 2. However, when slight discrepancies from $\theta_{theo}$ were introduced, some of the parameters reported by the optimal solution deviated considerably from their nominal value. Hence, in practice, it may not be possible to retrieve all five parameters simultaneously without a priori information to limit the variation range of said five parameters. It was found that the sensitivity of the solution to the random variation in angles was reduced when at least two of the parameters of the pipe and the liquid were provided as a priori information, thus reducing the degrees of freedom for which Equation (5) was being solved. Furthermore, to implement this method in practice, the two parameters provided a priori should be easy to obtain from other sources (e.g., by the operator in the field) or measured by the transducer arrays with sufficient accuracy. For these reasons, we proposed to provide the parameters $h_{pipe}$ and *D* as a priori information to Equation (5), and finally solve it to obtain $c_{pipe}$, *i* and $c_{liquid}$.

**Figure 5:**
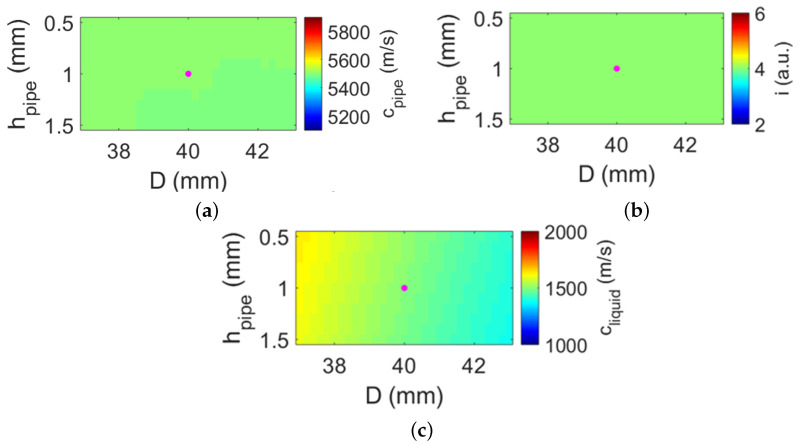
Parameters of the pipe and the liquid obtained from beam auto-alignment measurements and solving Equation (5) for (**a**) $c_{pipe}$, (**b**) *i*, and (**c**) $c_{liquid}$, using *D* and $h_{pipe}$ as a priori information. The range of the color scale of each map is centered around the nominal value (i.e., ground truth) of the parameter being obtained. The magenta dot on each map reports the coordinate associated with the nominal values of the a priori parameters (i.e., (D=40mm; $h_{pipe}=1\;\mathrm{mm}$)).

**Figure 6:**
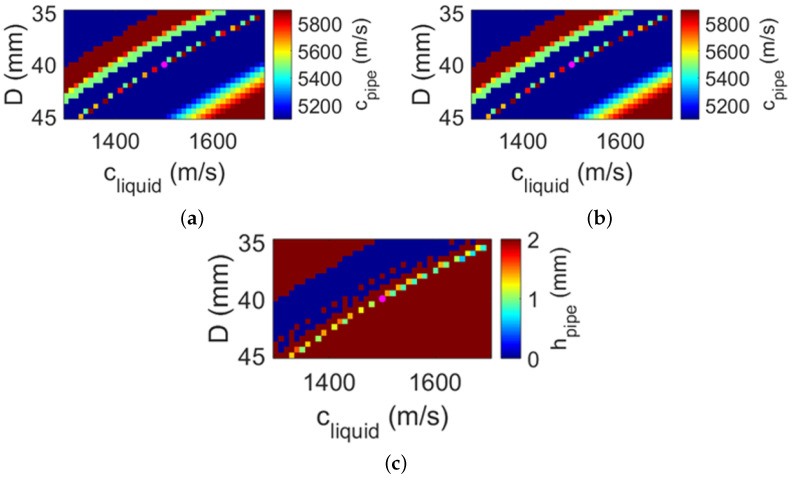
Parameters of the pipe and the liquid obtained from beam auto-alignment measurements and solving Equation (5) for (**a**) $c_{pipe}$, (**b**) *i*, and (**c**) $h_{pipe}$, using $c_{liquid}$ and *D* as a priori information. The range of the color scale of each map is centered around the nominal value (i.e., ground truth) of the parameter being obtained. The magenta dot on each map reports the coordinate associated with the nominal values of the a priori parameters (i.e., ($c_{liquid}=1500\;m/s$; D=40mm)).

Figure 5 shows the obtained pipe and liquid parameters using the measured optimal angles (i.e., $\theta_{meas}$ in Table 1) and solving Equation (5) for $c_{pipe},i,c_{liquid}$. The results shown in Figure 5 reflect the very low sensitivity of the solutions to the a priori parameters *D* and $h_{pipe}$, as desired for a practical implementation. In contrast, Figure 6 shows that the obtained solutions for $c_{pipe},i,h_{pipe}$ are very sensitive to the accuracy of the a priori (fixed) parameters $c_{liquid}$ and *D*, which is less desirable for practical applications. In Figure 5, the reported solutions for the nominal values D=40mm and $h_{pipe}=1\;\mathrm{mm}$ are $c_{pipe}=5498\;m/s$, i=4, and $c_{liquid}=1499\;m/s$, which correspond very well with the nominal values reported in Table 2. The value for $c_{pipe}$ differs by approximately 7% from the expected nominal value, which is comparable to discrepancies obtained with other methods reported in the literature [20]. Using these parameters in combination with Equation (1) would result in flow speed values with a discrepancy of less than 0.07% compared to flow speeds computed using the nominal parameters. This discrepancy is a much lower value compared to the 2.5% measurement error reported by commercially available clamp-on UFMs [1].

## 5. Discussion

Some sources of error in the results shown in the Section 4 may be: the steering angular step used to find the optimal angle of the acoustic beam for each travel path shown in Figure 4; (slight) misalignment between the pipe axis and the line intersecting the center points of the two arrays; and the acquisition system, which is not optimized for flow measurements and thus has a higher noise floor compared to typical flow metering systems.

Based on the obtained results, prior to flow metering, clamp-on UFMs based on transducer arrays may self-calibrate by performing the following steps: first, the pipe inner diameter (*D*) and wall thickness ($h_{pipe}$) are either provided as input values or measured by the sensor using techniques such as those proposed in [11,20]; second, electronic beam steering is used to find the optimal steering angle of the acoustic beam for three different travel paths; third, similar to Equation (5), a system of three equations with three unknown parameters of the pipe and the liquid (i.e., $c_{pipe},i,c_{liquid}$) is solved.

In practice, the time it would take for the sensor to find the optimal beam steering angle for each particular travel path of the acoustic beam would be proportional to several factors, such as the beam steering angular step used, the number of signals averaged, and the transit time of the acoustic beam. Thus, the speed of beam alignment measurements could be optimized by implementing the following: a relatively coarse beam steering angular step followed by an interpolation of the amplitude vs. angle curves; an adaptive angular step size based on the amplitudes of the measured signals; coded excitation to increase the single-shot SNR of the acoustic signals and thus reduce the amount of averages per angle; alignment of the acoustic beam for the three shortest travel paths since they would be associated with the shortest transit times and the highest SNRs. These optimal paths, in combination with the proposed method to extract the properties of the pipe and the liquid, would ultimately result in a lower measurement uncertainty compared to that of manually calibrated clamp-on UFMs.

For flow metering, the chosen travel paths of the acoustic beams of UFMs are those which are long enough so that the measured transit time differences are larger than the zero flow error of the measurement system. It would be good practice to also consider such travel paths for the calibration method proposed in our paper, especially because it may also be implemented during fluid flow.

Compared to the nonlinear algorithm that was used to solve Equation (5), a linear approximation of this equation would allow us to implement much simpler and faster solvers. The software Wolfram Mathematica (Wolfram Research, Champaign, IL, USA) was used to perform a Taylor series expansion of Equation (3) on the tan(·) term centered around the initial value for $c_{pipe}$ and $c_{liquid}$; the linear terms were kept and Equation (5) updated. Furthermore, using the theoretical angles shown in Table 1 for each travel path, the same software was used to numerically solve the linear version of Equation (5). The obtained solution corresponded to the nominal values of the parameters of the pipe and the liquid, indicating that a linearized version of Equation (5) could also be suitable for practical implementation.

Pipe wall thickness and diameter variations along the pipe axis are not uncommon in practice. These variations distort the travel path of the acoustic beam (i.e., the center of the generated beam does not coincide with the center of the receiver aperture), introducing errors in the computed flow speed. In contrast to current single-element clamp-on UFMs, a transducer array-based sensor would be able to compensate for such travel path distortions by performing electronic beam steering and automatically aligning the center of the acoustic beam with that of the receiver aperture. However, this effect is not accounted for in the method presented here when computing the flow speed, since Equation (1) uses one input value for *D* to compute $v_{f}$. In cases of highly attenuating liquids, in which SNR levels are very low and it is not possible to measure and align the acoustic beam for three travel paths, it is recommended to obtain the parameters of the pipe and the liquid by other methods, such as those based on the excitation and measurement of guided waves in the pipe wall [20].

Throughout the lifetime of clamp-on UFMs, pressure and temperature conditions of the pipe wall and the liquid/gas may vary from the conditions under which the sensor was first calibrated. Moreover, the liquid/gas flowing through the pipe may simply change. As a consequence, the properties of the pipe and the liquid relevant to flow metering may vary from the provided/measured initial values. Moreover, in practice, pipelines are usually exposed to aging conditions such as solar radiation, weather, and/or dust particles, which also modify the properties of the pipes, especially of non-metallic ones (e.g., PVC). These effects cause the acoustic beam to shift from its originally calibrated (aligned) travel path, and ultimately introduce an error in the flow speed. The electronic beam steering capability of transducer array-based clamp-on UFMs would allow us to dynamically re-calibrate the sensor by adjusting the steering angle of the acoustic beam to correctly align its center with that of the receiver aperture. Based on these adjusted optimal angles, Equation (5) may be solved to obtain updated values for the parameters of the pipe and liquid. This automated re-calibration technique may be implemented regularly throughout the lifetime of the flow meter. Moreover, compared to a manual calibration approach, which is usually operator-dependent, this automated technique would allow us to produce more repeatable results.

Although a cost analysis of the proposed array-based clamp-on UFM was outside of the scope of this paper, it is worthwhile to realize that, with increased functionality, the cost of array-based UFMs may be larger than that of current sensors. However, it is also important to note that, in terms of functionality, nothing beyond simple beam steering and beam forming operations is required from the arrays. These operations are already widely used in commercially available systems (such as in medical imaging and non-destructive testing applications). Thus, the total cost of the sensor development will be limited.

## 6. Conclusions

In this paper, it has been shown that clamp-on ultrasonic flow meters based on transducer arrays have the capability to automatically find the optimal travel path of the generated acoustic beam without the need to manually move the transducers along the pipe wall. Furthermore, using such beam auto-alignment measurements, a method to invert for the parameters of the pipe and the liquid required to perform a quantitative flow measurement has also been proposed. It consists of building and solving a system of equations based on the measured optimal beam steering angle of the acoustic beam through a few travel paths. The obtained values show very good agreement with the expected (nominal) values. Compared to a manual calibration approach, the ideas shown here would allow one to realize a user-independent self-calibrated clamp-on ultrasonic flow meter with more reliable and more repeatable results.

## Figures and Tables

**Table 1:** Theoretical and measured optimal angles ($\theta_{theo}$ and $\theta_{meas}$, respectively) for the five different travel paths of the acoustic beam shown in Figure 4.

*n*	$\theta_{\mathrm{theo}}$ (${}^{\circ }$)	$\theta_{\mathrm{meas}}$ (${}^{\circ }$)
4	17.91	17.90
5	14.87	14.95
6	12.64	12.65
7	10.98	10.80
8	9.70	9.50

**Table 2:** Nominal values of the parameters of the pipe and the liquid, and the values obtained from beam auto-alignment measurements and solution of Equation (5), for a priori values D=40mm and $h_{pipe}=1\;\mathrm{mm}$.

Parameter	Nominal Value	Measured Value
$c_{pipe}$	5920m/s	5498m/s
***i***	4	4
$c_{liquid}$	1500m/s	1499m/s

## Data Availability

Not applicable.

## Abbreviations

The following abbreviations are used in this manuscript:

UFMs	Ultrasonic flow meters
SNR	Signal-to-noise ratio
*mad*	Mean absolute deviation

## Appendix A. Derivation of the Equation to Compute Flow Speed Considering an Ultrasonic Flow Meter in a Clamp-On Configuration

Considering an acoustic beam during clamp-on ultrasonic flow metering, such as the one shown in Figure 2, the length *L* of its travel path is:

(A1) $$\begin{array}{c} L=L_{cp}+L_{pipe}+L_{liquid}, \end{array}$$

with $L_{cp},L_{pipe},L_{liquid}$ being the length of the travel path of the beam in the coupling piece, the pipe wall, and the liquid, respectively.

The upstream and downstream transit time *T* of the acoustic beam is then given by:

(A2) $$\begin{array}{c} T_{u,d}=\frac{L_{cp}}{c_{cp}}+\frac{L_{pipe}}{c_{pipe}}+\frac{L_{liquid}}{c_{liquid}\mp v_{f}\sin\beta }, \end{array}$$

where $c_{cp}$, $c_{pipe}$, and $c_{liquid}$ are the wave sound speeds of the coupling piece, of the pipe wall, and of the liquid, respectively; $v_{f}$ represents the flow speed, and β represents the refraction angle, relative to the normal of the pipe wall–liquid interface (see Figure 2), of the acoustic beam into the liquid. The ∓ sign points out the upstream (−) and downstream (+) case.

The transit time difference Δt between both transit times is then:

(A3) $$\begin{array}{cc} \Delta t & =T_{u}-T_{d}\\ \; & =L_{liquid}\left(\frac{1}{c_{liquid}-v_{f}\sin\beta }-\frac{1}{c_{liquid}+v_{f}\sin\beta }\right). \end{array}$$

From the geometry in Figure 2, $L_{liquid}$ is:

(A4) $$\begin{array}{c} L_{liquid}=\frac{2nD}{\cos\beta }, \end{array}$$

where *n* represents the number of bounces (i.e., v-shaped reflections) of the acoustic beam within the pipe, and *D* represents the inner diameter of the pipe.

Substituting Equation (A4) into Equation (A3):

(A5) $$\begin{array}{c} \Delta t=\left(\frac{2nD}{\cos\beta }\right)\left(\frac{1}{c_{liquid}-v_{f}\sin\beta }-\frac{1}{c_{liquid}+v_{f}\sin\beta }\right). \end{array}$$

Finally, after multiplication and factorization in Equation (A5), the following second-order equation for the flow speed $v_{f}$ results:

(A6) $$\left[\Delta t\sin^{2}\left(\beta \right)\right]v_{f}^{2}+\left[4nD\tan\left(\beta \right)\right]v_{f}-\Delta tc_{liquid}^{2}=0.$$

The solutions for $v_{f}$ are:

(A7) $$v_{f}=\left(\frac{-b\pm \sqrt{b^{2}-4ac}}{2a}\right),$$

with:

(A8) $$a=\Delta t\sin^{2}\left(\beta \right),$$

(A9) b=4nDtan(β),

(A10) $$c=-\Delta tc_{liquid}^{2}.$$

Since $b^{2}-4ac>0$ in Equation (A7), both roots of the equation are real numbers. One is positive, and the other is negative. For a physically valid solution, the positive root is chosen as the solution to Equation (A7).

At relatively low flow speeds, i.e., when $c_{liquid}\gg v_{f}$, the nonlinear term of Equation (A6) may be discarded. However, this term becomes more relevant in cases where the flow speed becomes comparable to the sound speed of the liquid [6].
